# Mechanisms and Determinants of Ultralong Action Potential Duration and Slow Rate-Dependence in Cardiac Myocytes

**DOI:** 10.1371/journal.pone.0043587

**Published:** 2012-08-27

**Authors:** Zhilin Qu, Douglas Chung

**Affiliations:** Department of Medicine (Cardiology), David Geffen School of Medicine, University of California Los Angeles, Los Angeles, California, United States of America; University of Illinois at Urbana-Champaign, United States of America

## Abstract

In normal cardiac myocytes, the action potential duration (APD) is several hundred milliseconds. However, experimental studies showed that under certain conditions, APD could be excessively long (or ultralong), up to several seconds. Unlike the normal APD, the ultralong APD increases sensitively with pacing cycle length even when the pacing rate is very slow, exhibiting a sensitive slow rate-dependence. In addition, these long action potentials may or may not exhibit early afterdepolarizations (EADs). Although these phenomena are well known, the underlying mechanisms and ionic determinants remain incompletely understood. In this study, computer simulations were performed with a simplified action potential model. Modifications to the L-type calcium current (I_Ca,L_) kinetics and the activation time constant of the delayed rectifier K current were used to investigate their effects on APD. We show that: 1) the ultralong APD and its sensitive slow rate-dependence are determined by the steady-state window and pedestal I_Ca,L_ currents and the activation speed and the recovery of the delayed rectifier K current; 2) whether an ultralong action potential exhibits EADs or not depends on the kinetics of I_Ca,L_; 3) increasing inward currents elevates the plateau voltage, which in general prolongs APD, however, this can also shorten APD when the APD is already ultralong under certain conditions; and 4) APD alternans occurs at slow pacing rates due to the sensitive slow rate-dependence and the ionic determinants are different from the ones causing APD alternans at fast heart rates.

## Introduction

**Figure 1:**
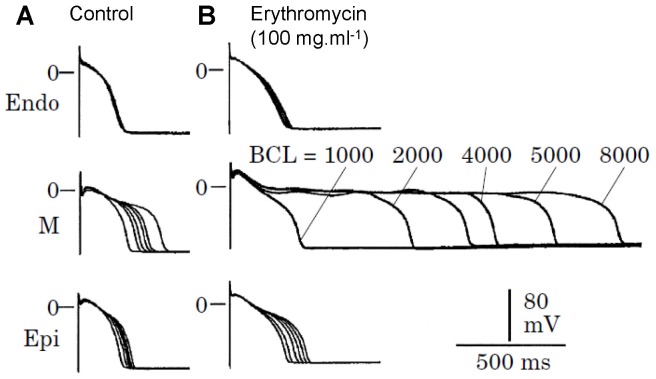
Effects of I_Kr_ blocker enrythromycin on APD and rate-dependence in myocytes from epicardial (Epi), midmyocardial (M), and endocardial (endo) of canine left ventricle (reproduced from Antzelevitch [**5**]).

Under normal conditions, the action potential duration (APD) of a ventricular myocyte ranges from less than 100 ms (such as in mice or rats) to around 300 ms (such as in large animals or human). In diseased conditions and/or the presence of drugs [1]–[8], APD can be much longer, reaching several seconds (ultralong), or even fail to repolarize. It is well known that APD is a result of the balance between the inward and outward currents, lengthening as inward currents increase or outward currents decrease, and vice versa. In many cases, lengthening of APD by increasing inward currents or decreasing outward currents leads to early afterdepolarizations (EADs) [1], [2], [3], which are voltage oscillations during the repolarizing phase of the action potential. For example, BayK8644, which increases the open probability of the L-type calcium (Ca) channel (LCC), or E4031, which blocks the rapid component of the delayed rectifier potassium (K) current (I_Kr_), lengthens APD and promotes EADs [1], [9]. But in other cases, lengthening APD does not lead to EADs even though APD can be as long as seconds. For example, blocking I_Kr_ by either erythromycin (Fig. 1) or quinidine lengthens APD up to 2 s without inducing EADs in the midmyocardial cells (M-cells) [4], [5]. Expression of wild type β_2a_ subunit of LCC lengthens APD and causes EADs, while mutations of the β_2a_ subunit lacking the CaMKII phosphorylation sites lengthen APD to more than 2 s without causing EADs [6]. Overexpression of Ca-insensitive calmodulin mutant (CaM_1234_) in rabbit ventricular myocytes to inhibit the Ca-dependent inactivation of LCC also lengthens APD to 2 s without inducing EADs [7], [8]. This raises two questions: 1) which ionic currents or ion channel properties are responsible for the ultralong APDs? And 2) why does lengthening APD by increasing inward currents or decreasing outward currents induce EADs in some cases but not in other cases? Another interesting observation in the experiments by Antzelevitch and colleagues [4], [5] is that the M-cells exhibit a much slower APD rate-dependence than the epicardial and endocardial cells. After I_Kr_ is blocked by erythromycin or quinidine, the APD rate-dependence is further enhanced (Fig. 1). This raises another question: why does the APD of the M-cells exhibit sensitive slow rate-dependence but the epicardial or endocardial cells do not?

Answering these questions is important for understanding arrhythmogenesis in cardiac tissue and developing effective anti-arrhythmic therapeutics. For example, lengthening APD without EADs may be less arrhythmogenic than that with EADs since EADs can cause premature ventricular complexes which are arrhythmia triggers. In addition, the presence of EADs may also promote larger dispersion of refractoriness. This was addressed in our recent study [10], in which we showed that in the presence of random cell-to-cell variations, large APD dispersion could form when EADs were present in the action potentials due to the “all-or-none” property of EAD occurrence. But large cell-to-cell variations can be effectively smoothed by gap junction coupling when EADs were absent, resulting in a small APD dispersion.

Many modeling studies have been carried out on the genesis of EADs in ventricular myocytes [11]–[18], which have gained deep insight into the roles of different ionic currents or genetic defects in promoting or suppressing EADs. In recent studies [19], [20], we have shown that EADs are caused by a dual Hopf-homoclinic bifurcation, which provides a unifying dynamical mechanism for EADs in cardiac myocytes. However, the aforementioned questions have not been properly addressed in the previous modeling studies.

In this study, we use mathematical modeling and theoretical analysis to address these questions. To facilitate theoretical analysis and sort out the minimal requirements, we used a simplified action potential model, the 1991 Luo and Rudy (LR1) model [21]. We show that: 1) the ultralong APD and its slow and sensitive rate-dependence are determined by the window and pedestal L-type Ca current (I_Ca,L_) currents and the activation and recovery speed of the delayed rectifier K current; 2) whether the action potential exhibits EADs or not depends on the kinetics of I_Ca,L_, such as the slope of the steady-state inactivation curve; 3) increasing inward currents in general prolongs APD but can shorten APD when APD is already ultralong under certain conditions; and 4) APD alternans occur at slow pacing rates due to the sensitive slow rate-dependence, a new mechanism of alternans different from fast pacing induced APD alternans, which may be responsible for T-wave alternans occurring at normal or slow heart rates in clinical settings, such as long QT syndrome [22], [23].

## Methods

**Figure 2:**
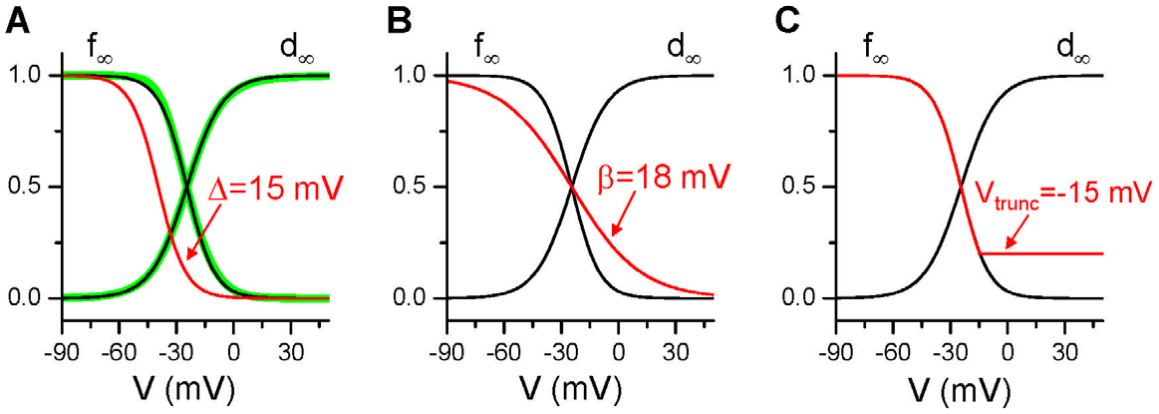
Alterations of I_Ca,L_ kinetics. The black lines are d_∞_ and f_∞_ form Eq.3 with β = 7.2 and Δ = 0. The thick green lines in A are the ones from the original LR1 formulation. The red line in each panel is the altered f_∞_. **A**. Δ = 15 mV. **B**. β = 18. **C**. V_trunc_ = −15 mV.


The differential equation governing the membrane voltage (V) is:
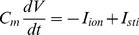
(1)where C_m_ = 1 µF/cm^2^ is the membrane capacitance, I_ion_ the total ionic current density of the LR1 model, and I_sti_ the stimulus current density with a strength of 25 µA/cm^2^ and a duration of 2 ms. In the LR1 model, the plateau inward current is called slow inward current (I_si_) which is the same as the L-type Ca current (I_Ca,L_, a notion will be used in this study), which was formulated as



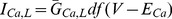
(2)To alter the window and pedestal I_Ca,L_, we used the following functions for d_∞_ and f_∞_:
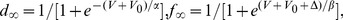
(3)while the activation time constant τ_d_ and inactivation time constant τ_f_ were unchanged from the original formulations. When V_0_ = 24.5 mV, α = 9.4 mV, β = 7.2 mV, and Δ = 0, d_∞_ and f_∞_ are very close to the original curves (the green lines in Fig. 2). In this study, Δ was used to shift the inactivation curve to alter the window current (Fig. 2A). β was used to change the slope of the inactivation curve (Fig. 2B). To model the pedestal current or incomplete inactivation, we truncated f_∞_ as follows: we chose a voltage V_trunc_ and set f_∞_ to its value at V_trunc_ if V>V_trunc_ (Fig. 2C). In the LR1 model, there is only one time-dependent K current (I_K_), whose formulation is

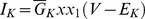
(4)

This current is equivalent to the slow component of the delayed rectifier K current (I_Ks_) [24], [25]. However, the slow activation time constant of I_Ks_ was measured to be longer than 2 s [25], which is much longer than the time constant (τ_x_) in the LR1 model (∼600 ms at −20 mV). To study the effects of the activation speed of I_K_, we multiplied τ_x_ by a factor γ, i.e., τ_x_(V)→γτ_x_(V), to alter its activation speed.

Eq.1 was numerically solved using a fourth-order Runge-Kutta method with a time step 0.01 ms. APD is defined as the time duration during which V>−72 mV and diastolic interval (DI) is defined as the time duration during which V<−72 mV.

## Results

### Roles of Different Ionic Currents in Generating Ultralong APD and EADs

#### Window ICa,L

**Figure 3:**
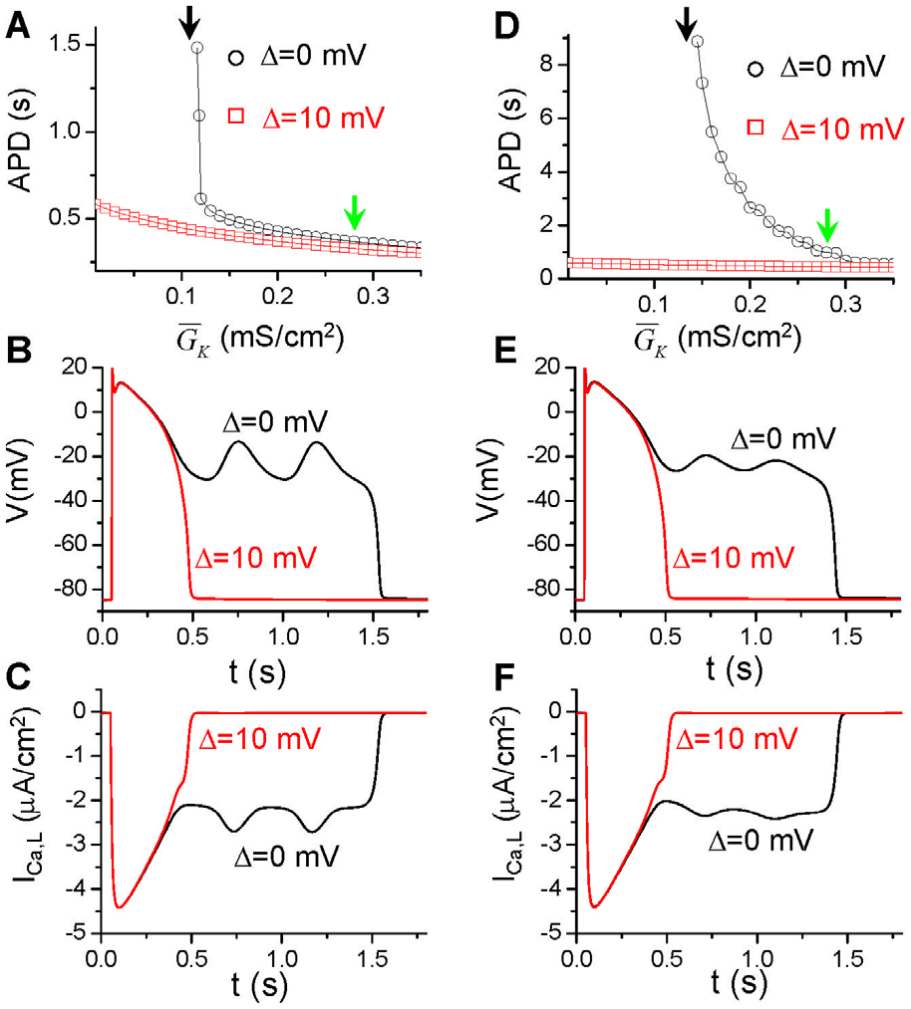
Effects of window I_Ca,L_ and activation speed of I_K_ on APD. **A**. APD vs 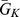 from the original LR1 model and from the one with 10 mV shift in f_∞_ (Δ = 10 mV). **B**. Two action potentials for 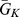 = 0.116 mS/cm^2^ in the original LR1 model and the one with 10 mV shift in f_∞_. **C**. I_Ca,L_ during the two action potentials in B. **D**-**F**. Same as A-C but for γ = 4. 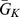 = 0.25 mS/cm^2^ for E and F. Black arrows in A and D indicate that repolarization failure occurs when 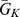 is smaller than these values in the case of Δ = 0 mV (open circles). Green arrows indicate the control 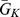 (0.282 mS/cm^2^). The voltage was initially set to −84 mV with other variables close to their steady states. After 10 s (t = 0 in the plots), a single stimulus was given to elicit an action potential. The same stimulation protocol was used for Figures 4 and 5.

In the original LR1 model (circles in Fig. 3A), reducing the maximum conductance (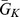) of I_K_ lengthens APD gradually until 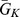
is reduced to 0.12 mS/cm^2^ after which APD increases abruptly to very large values in a narrow 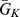 range (between 0.115 mS/cm^2^ and 0.12 mS/cm^2^). Repolarization failure occurs when 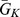
is smaller than 0.115 mS/cm^2^. In the long action potentials, EADs occur (solid line in Fig. 3B). To study how window I_Ca,L_ affect APD and the occurrence of EADs, we shifted the steady-state inactivation curve (f_∞_) of I_Ca,L_ by 10 mV to more negative voltage (Δ = 10 mV), which reduces the window I_Ca,L_. This shift eliminates the ultralong APD and EADs even when 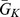 is reduced to zero (squares in Fig. 3A). Shifting f_∞_ by 10 mV to more negative voltages decreases APD slightly from the control until 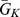 is reduced to a value at which ultralong APD and EADs occur. In Figure 3B, we also plot the action potential for the same 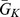
(0.116 mS/cm^2^) after f_∞_ is shifted (dashed line), showing that the voltage traces before and after the shift are almost identical in the first several hundred milliseconds until voltage decreases to around −20 mV in the repolarization phase at which the two traces go apart. Figure 3C plots the corresponding I_Ca,L_ traces, showing that shifting f_∞_ by 10 mV has almost no effect on I_Ca,L_ until the voltage decreases to around −20 mV at which window I_Ca,L_ becomes important. Note that it is well known that window I_Ca,L_ is important for EAD genesis [26], [27] and the effects of shifting the inactivation kinetics on EAD genesis have been demonstrated in dynamic clamp experiments by Madhvani et al [28].

**Figure 4:**
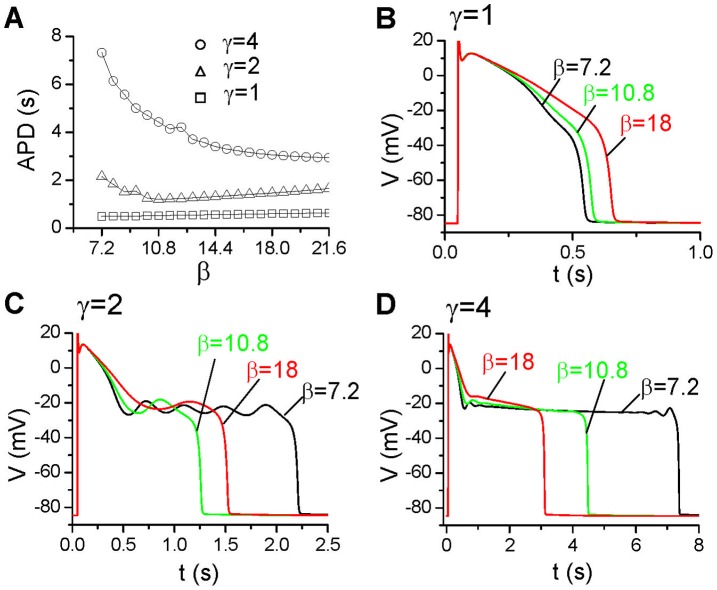
Effects of reduced f_∞_ slope. **A**. APD vs β for different γ. 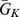 = 0.15 mS/cm^2^. **B**-**D**. Action potentials for β = 7.2, 10.8, and 18 for the γ = 1(B), γ = 2(C), and γ = 4 (D).

**Figure 5:**
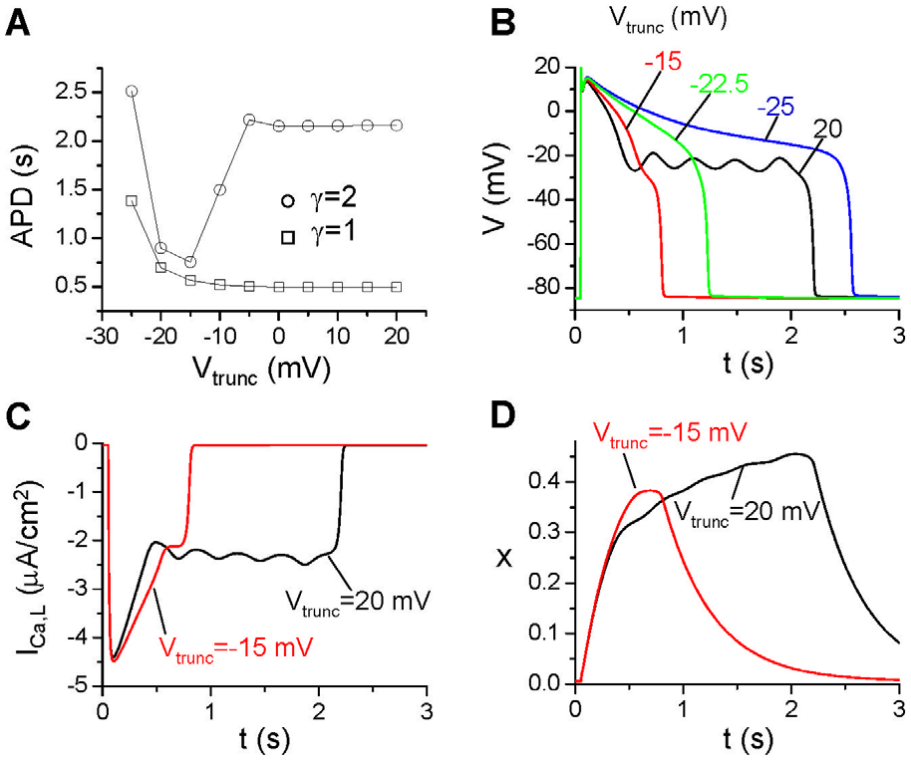
Effects of pedestal I_Ca,L_. **A**. APD vs V_trunc_ for different γ. 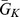 = 0.15 mS/cm^2^. **B**. Action potentials for different V_trunc_ for γ = 2. **C**. I_Ca,L_ vs time for V_trunc_ = 20 mV and −15 mV. **D**. The activation gate x of I_K_ vs. time for the two action potentials in C.

#### Speed of IK activation

Slowing the activation of I_K_ lengthens APD, and makes the long APD region much wider. Figure 3D shows APD versus 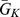
for I_K_ activation slowed 4 times (γ = 4), ultralong APD exists between 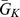 = 0.14 mS/cm^2^ and 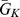 = 0.28 mS/cm^2^ (see circles in the upper panel in Fig. 3D) and EADs occur in the long action potentials. However, shifting the inactivation curve of I_Ca,L_ 10 mV to more negative voltages eliminates the occurrence of the ultralong action potentials (squares in the upper panel in Fig. 3D). Shifting inactivation curve to reduce the window I_Ca,L_ has almost no effect on voltage in the first several hundred milliseconds of the action potential (Fig. 3E) as well as peak I_Ca,L_ (Fig. 3F).

#### Slope of the steady-state inactivation curve of ICa,L

The results shown in Figure 3 indicate that the height of the window I_Ca,L_ and the activation speed of I_K_ are important determinants of long APD and EADs. To further study the effects of I_Ca,L_ properties on APD, we reduced the slope of its inactivation curve (see Fig. 2B). In Figure 4A, we show APD versus the slope factor (β) for 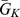 = 0.15 mS/cm^2^ and three different I_K_ activation speeds. When γ = 1 where no EADs occur in the action potential, decreasing the slope of the inactivation curve increases APD, with action potentials for three different slopes shown in Figure 4B. When the activation of I_K_ is slower (γ = 2), APD decreases first and then increases as the slope decreases. In Figure 4C, we show the action potentials for three different slopes: for β = 7.2 (the original slope), four EADs occur in the action potential; for β = 10.8, the action potential becomes shorter with only one small EAD; and for β = 18, the action potential becomes longer again with only one small EAD. When the activation of I_K_ is even slower (γ = 4), APD decreases as β increases. When β = 7.2, small voltage oscillations occur when the voltage first decreases to −20 mV and then disappear and reappear before repolarizing to the resting potential (Fig. 4D). But for β = 10.8 and β = 18, there are no voltage oscillations in the plateau phase (no EADs). The ultralong APD can also be eliminated by shifting f_∞_ to more negative voltages.

#### Pedestal ICa,L

Besides the steady-state current in the window range, I_Ca,L_ also exhibits a pedestal component [29] due to incomplete inactivation of the channel. To study how this component affects the action potential properties, we truncated f_∞_ as described in the Methods section (see Fig. 2C for an example). The reason for changing the function like that is to maintain the same window current (no change in the height of the window) with incomplete inactivation to generate a pedestal current. In Figure 5A, we show APD versus V_trunc_ for γ = 2, showing that APD remains unchanged when V_trunc_ is in the positive voltage range but then decreases as V_trunc_ decreases. After reaching a minimum, APD increases again. When V_trunc_ is in the positive voltages, the truncated f_∞_ is almost the same as the original one, and thus APD changes little with V_trunc_, and EADs occur in the action potential (Fig. 5B). Interestingly, as V_trunc_ decreases, the pedestal I_Ca,L_ increases, however, the APD decreases and EADs disappear. Although as V_trunc_ decreases further, APD increases again and becomes ultralong, no EADs occur in the action potential. As shown in Figure 5C, I_Ca,L_ is bigger for V_trunc_ = −15 mV than for V_trunc_ = 20 mV in the early repolarizing phase, which is responsible for the higher plateau voltage for V_trunc_ = −15 mV shown in Figure 5B. This is contradictory to the traditional understanding that increasing inward currents increases APD and promotes EADs.

### Mechanistic Insight into Ultralong APD and EAD Genesis

**Figure 6:**
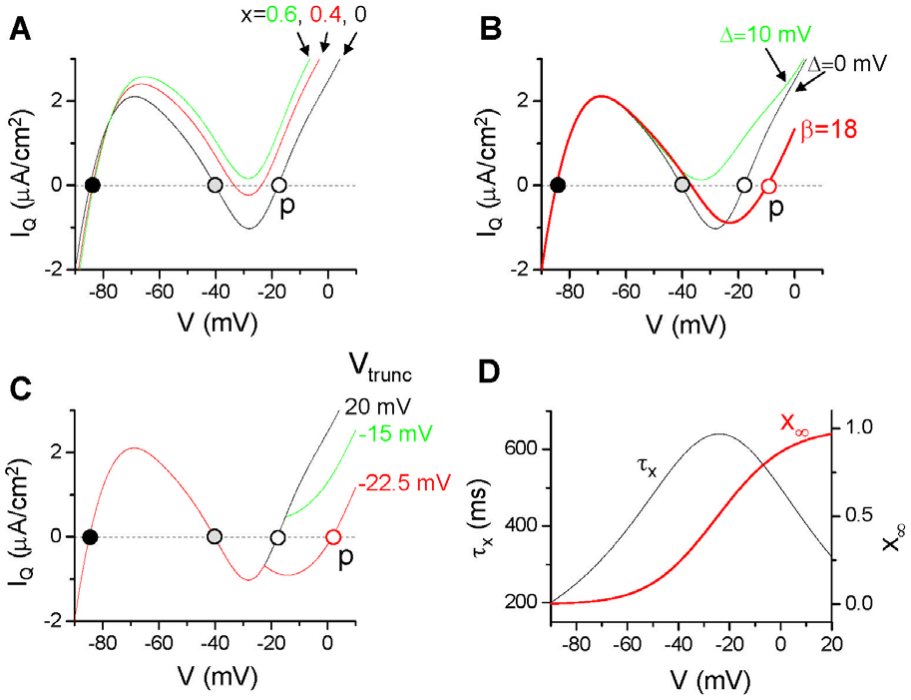
Quasi-steady state I-V curves. **A**. The quasi-steady state whole-cell current (I_Q_) vs. voltage in the original model with 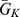 = 0.15 mS/cm^2^ with x set at different constant values (as marked). **B**. I_Q_ vs. voltage with x = 0 for the original model (Δ = 0), 10 mV shift in f_∞_ (Δ = 10 mV), and f_∞_ slope reduced (β = 18). **C**. I_Q_ vs. voltage with x = 0 for different V_trunc_. **D**. Activation time constant (τ_x_) and the steady-state action curve (x_∞_) of I_K_ vs. voltage from the original LR1 model.

As shown above, the interactions between inward currents and outward currents exhibit complex effects on affecting APD and the occurrence of EADs. To better understand the underlying mechanisms, we plot the quasi-steady state (or quasi-instantaneous, a notion used in [7], [30]) total current versus voltage (quasi-steady state I-V curve) in Figure 6, which is the steady-state whole-cell current (the I_ion_ in Eq.1) with the activation gating variable x of I_K_ (see Eq.4) fixed at a constant value. This type of plots was used by Noble [30] and Alseikhan et al [7] to explain all-or-none repolarization and ultralong APD. It was also widely used in the FitzHugh-Nagumo type models to study the fast-slow dynamics [31], [32]. Figure 6A shows the quasi-steady state I-V curves when the x values are set as different constants. When x = 0, there are three voltages (circles in Fig. 6A) at which the quasi-steady state current is 0 (i.e., the total outward current equals to the total inward current), we call them quasi-equilibrium states. The one between −90 mV and −80 mV is the resting potential. As x increases, the quasi-steady state I-V curve moves upward, and when x becomes large (e.g., >0.5 in Fig. 6A), the upper two quasi-equilibrium states disappear and only the equilibrium state for the resting potential remains. Using the quasi-steady state I-V curves, we can gain mechanistic insights into the roles of different ionic currents on APD and EAD genesis as follows.

#### Activation speed and conductance of IK

At the beginning of an action potential, x is small. As the voltage quickly increases from the resting potential to the positive voltage range, x begins to increase, and the voltage is approaching the upper quasi-equilibrium state (the open circle in Fig. 6A). As x grows to large values (e.g., x>0.5), the quasi-equilibrium states (the gray and open circles in Fig. 6A) at the plateau voltage disappear, and the voltage then decreases quickly to the resting potential due to the large outward current (mainly I_K1_), repolarizing the cell. Since APD is determined by the time of the voltage staying at the plateau phase, the longer can the quasi-equilibrium state at the plateau voltage hold, the longer the APD is. Therefore, for a given conductance of I_K_, a slower activation speed (i.e., x grows slower) results in a longer existence of the quasi-equilibrium state. This maintains the voltage at the plateau phase for a longer time, resulting in a longer APD. As shown in Figure 3, the effect of slowing I_K_ activation on lengthening APD also depends on the conductance of I_K_. When the conductance is large, slowing activation has only a small effect on lengthening APD. When the conductance becomes smaller, the effect becomes more prominent, until the quasi-equilibrium state becomes a true equilibrium state at which repolarization fails.

#### Window ICa,L

The existence of the quasi-equilibrium states at the plateau voltage requires the presence of window I_Ca,L_ and its balance with the total outward current. If one shifts the I_Ca,L_ inactivation curve to more negative voltages to reduce or eliminate the window current, there will be no quasi-equilibrium states in the plateau voltage (e.g., 10 mV shift in f_∞_ eliminated the quasi-equilibrium states in Fig. 6B). Without the existence of the quasi-equilibrium states, the voltage cannot be held long in the plateau phase. Therefore, the presence of the window I_Ca,L_ is required for the occurrence of ultralong APD. As shown in Figure 3, a 10 mV shift in f_∞_ eliminates the ultralong APD even for 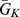 = 0 due to the elimination of the quasi-equilibrium states at the plateau voltage.

#### Pedestal ICa,L

Increasing the pedestal I_Ca,L_ by truncating f_∞_ moves the quasi-equilibrium state to higher voltages (from the dashed circle to the solid circle in Fig. 6C). However, the steady-state activation curve (x_∞_) of I_K_ increases with voltage and its activation time constant also depends on voltage, which becomes the largest around −20 mV (Fig. 6D). Therefore, increasing the pedestal I_Ca,L_ current shifts the quasi-equilibrium state to higher voltage which activates more I_K_ with a faster activation speed (see the example shown in Fig. 5D), making the quasi-equilibrium state to disappear even faster than without the pedestal current. Therefore, even though the pedestal I_Ca,L_ is an inward current, increasing it may shorten APD if the APD is already long in the absence of the pedestal current. However, as the pedestal I_Ca,L_ is increased further, it overcompetes the effects of faster activation of I_K_, prolonging APD again. This explains the observation shown in Figure 5.

#### Slope of the steady-state inactivation curve of ICa,L

Reducing the slope of f_∞_ increases the pedestal I_Ca,L_ which shifts the quasi-equilibrium state to higher voltages (Fig. 6B). Therefore, similar to truncating f_∞_, reducing the slope of f_∞_ results in a higher plateau voltage, which then activates more I_K_ with a faster activation speed, resulting in APD shortening as shown in Figure 4. Besides this effect, changing the slope of the inactivation curve also alter the stability of the quasi-equilibrium state at the plateau voltage. As shown in our previous study [19], the stability of the quasi-equilibrium state depends on many factors, e.g., the slopes of steady-state activation and inactivation curves and the activation and inactivation time constants of I_Ca,L_. When these parameters are properly set, the quasi-equilibrium state becomes unstable via a Hopf bifurcation, leading to voltage oscillations to manifest as EADs. As shown in Figure 4, when the slope of f_∞_ is reduced, either EADs disappear or their amplitudes are reduced, agreeing with the previous theoretical predictions [19].

### Slow Rate-dependence of APD

**Figure 7:**
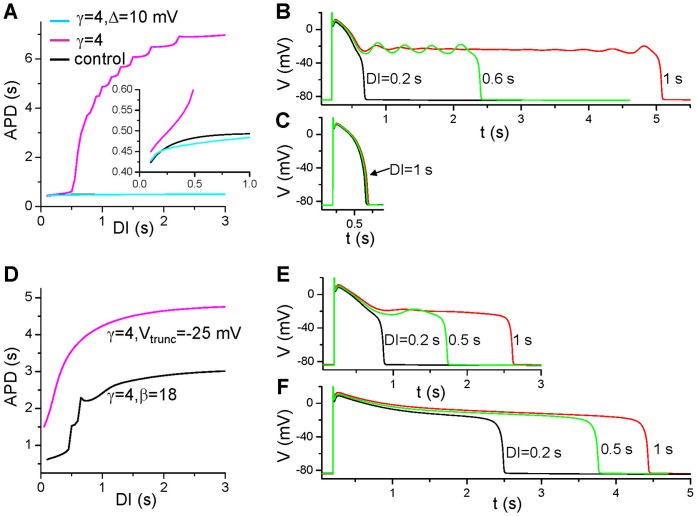
S1S2 APD restitution curves. **A**. APD vs. DI for the original model with 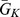 = 0.15 mS/cm^2^. γ = 1 (control, black line); γ = 4 (magenta); and γ = 4 and Δ = 10 mV (cyan). **B** and **C**. Action potentials at different DIs for γ = 4 and Δ = 0 mV (B) and Δ = 10 mV (C). **D**. APD vs. DI for β = 18 and γ = 4 (black), and for V_trunc_ = −25 mV and γ = 4 (magenta). **E**. Action potentials at different DIs for β = 18 and γ = 4. **F**. Action potentials at different DIs for V_trunc_ = −25 mV and γ = 4. A single S1 was given the same way as in Figures 3, 4, and 5 and an S2 was given at different S1S2 coupling intervals to study APD rate-dependence.

To study what causes the slow rate-dependence, we show in Figure 7 S1S2 APD restitution curves (APD versus DI) under different conditions. Figure 7A shows three APD restitution curves: 1) the original LR1 model (solid line); 2) slowed τ_x_ (dashed line); and 3) slowed τ_x_ and shifted f_∞_ (dashed-dotted line). Slow and sensitive rate-dependence occurs in the presence of window I_Ca,L_ and slowed I_K_ deactivation. Note that EADs occur in the long action potentials, but the amplitudes of EADs are smaller for longer DI (Fig. 7B). This can be explained by our previous theoretical analysis that the quasi-equilibrium state is more stable when x is smaller [19]. For example, for DI = 1 s, x is recovered to smaller values so that quasi-equilibrium state becomes stable at these x values, therefore, no voltage oscillations for a long period of time in the plateau phase. But as x glows, the quasi-equilibrium state becomes unstable, voltage oscillations occur, which gives rise to the EADs seen in the very end of the plateau (Fig. 7B). For DI = 0.6 s, x is less recovered and the quasi-equilibrium state is unstable, the voltage oscillations remain from the beginning to the end of the plateau. For either fast I_K_ deactivation or small window I_Ca,L_, APD cannot be prolonged to the ultralong ones and no EADs can occur (Fig. 7C) at any DI. For the cases of reduced f_∞_ slope and increased pedestal I_Ca,L_, slow and sensitive rate-dependence also occurs (Fig. 7D) but no EADs in the action potentials (Figs. 7E and 7F). The underlying mechanism of sensitive rate-dependence is the same as in the first case, but the quasi-equilibrium state is stable, and thus no EADs occur. Note that the action potentials shown in Figure 7E and the rate-dependence are similar to those shown in Figure 1 for the M-cells with I_Kr_ blocked.

### APD Alternans at Slow Pacing Rates

**Figure 8:**
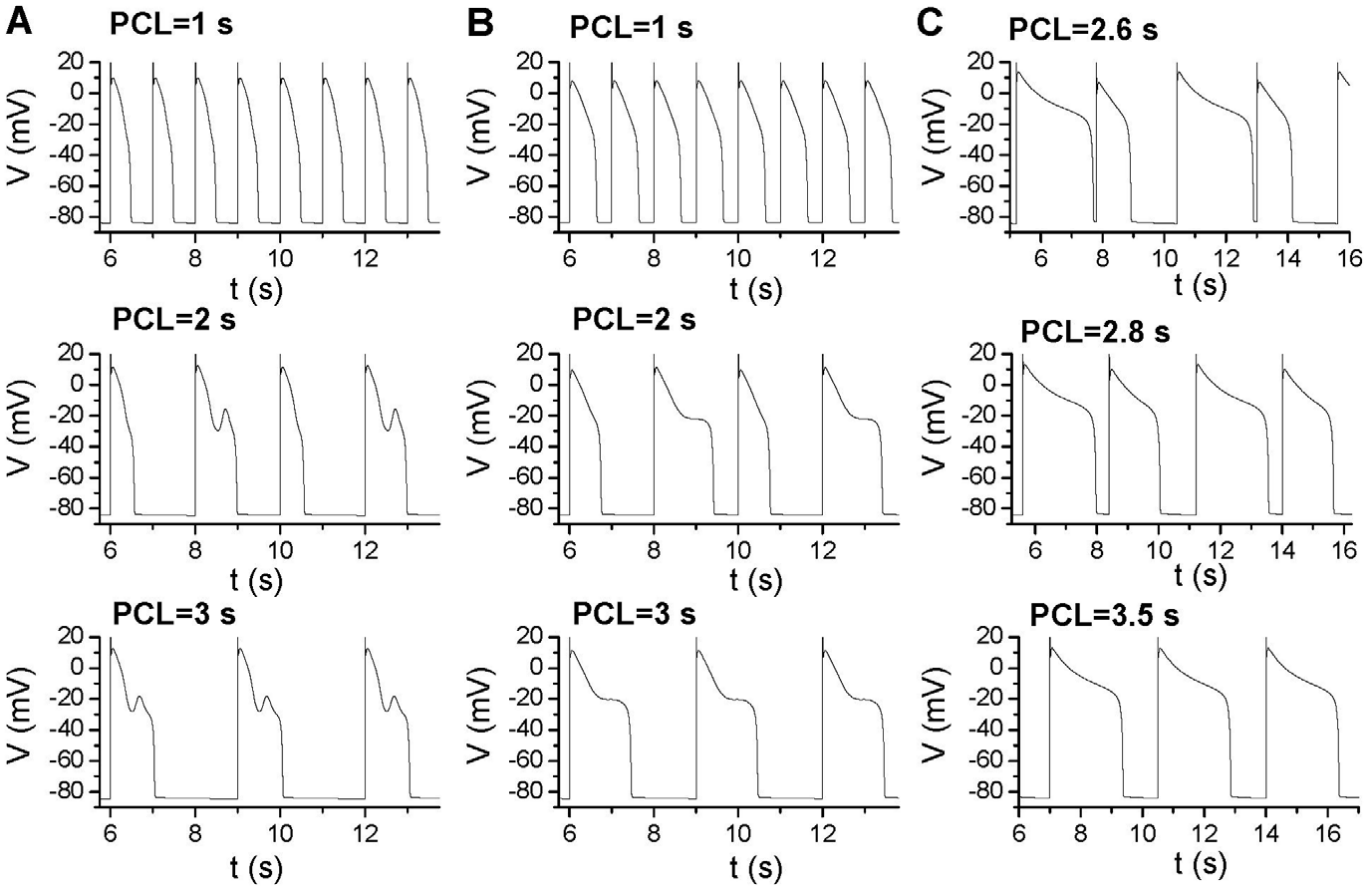
APD alternans at slow pacing rates. **A**. Action potentials for the original model with 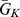 = 0.25 mS/cm^2^ and γ = 4 for three PCLs. **B**. Same as A but for a reduced slope of f_∞_ (β = 21.6) with 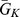 = 0.2 mS/cm^2^ and γ = 4. **C**. Same as A but for a truncated f_∞_ (V_trunc_ = −25 mV) with 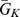 = 0.18 mS/cm^2^ and γ = 4.

As shown in Figure 7A, the sensitive APD changes occur at large DIs but not small DIs. This causes APD alternans to occur at slow pacing rates. For the original model with slowed activation of I_K_ (Fig. 8A), no alternans and EADs occur when PCL = 1 s. At PCL = 2 s, APD alternans occurs with every other action potential exhibiting an EAD. At PCL = 3 s, no APD alternans occurs while every action potential exhibits an EAD. The mechanism of alternans is due to the all-or-none behavior of EADs and steep APD restitution curve as we showed previously [22], [33]. For the case of reduced f_∞_ slope (Fig. 8B), alternans also occurs at PCL = 2 s but not at fast (e.g., PCL = 1 s) and slow (e.g., PCL = 3 s) pacing rates. In this case, no EAD is present in the action potentials. For the case of increased pedestal I_Ca,L_ (Fig. 8C), APD alternans also occurs at rates faster than PCL = 3.5 s until 2∶1 block occurs. The difference between this case and the previous two cases is that the sensitive APD changes occur at smaller DIs (see Fig. 7D), therefore, alternans remains until 2∶1 block.

## Discussion

Under normal conditions, APD in cardiac myocytes of large animals is 200–300 ms, which is mainly determined by I_Ca,L_, I_Ks_, I_Kr_, and I_K1_, with other currents playing smaller roles [34], [35]. I_Ca,L_ is the main inward current to act against the outward currents to maintain the plateau phase of the action potential, whose inactivation time constant is around 100 ms [29]. Therefore, without the presence of the steady-sate window or pedestal components, I_Ca,L_ will be completely inactivated within several hundred milliseconds, and no inward current is available to maintain the plateau voltage to result in a ultralong APD. To result in seconds long APD, very slow inactivating or steady-state inward currents are needed to form a quasi-equilibrium state at the plateau voltage. As we show in this study, the window and pedestal I_Ca,L_ are the candidate ones. Window I_Ca,L_ is also well known to be important for EAD formation [26], [27], [28]. Ca channel Ca_v_1.2 α subunit mutations, which are associated with type 8 long QT syndrome (or Timothy syndrome) [36], shift the activation and inactivation curves to increase both the window and pedestal I_Ca,L_ and cause APD lengthening and EADs to result in arrhythmias in Timothy syndrome. Our present study provides a clear mechanistic explanation of the roles of window and pedestal I_Ca,L_ in APD lengthening and EAD promotion, i.e., the presence of window or pedestal I_Ca,L_ is required to form a quasi-equilibrium state at the plateau voltage to generate long APD. But the occurrence of EADs is determined by the stability of the quasi-equilibrium state, which is mainly determined by the activation and inactivation kinetics and time constants of I_Ca,L_ [19]. These insights may help us to understand why BayK8644 causes EADs while CaM_1234_ does not. It has been shown that BayK8644 increases the window I_Ca,L_ and shifts the activation and inactivation curve to more negative voltages (with no changes in slopes of these curves) [37], [38]. Therefore, the major role of BayK8644 is to increase the window I_Ca,L_. However, CaM_1234_ alters the slope of the inactivation of I_Ca,L_ and increases the pedestal current [7]. Based our analysis, these properties tend to suppress EADs while result in ultralong APDs.

Besides the window and pedestal I_Ca,L_, other inward currents, such as late I_Na_ or window I_Na_ can also be the candidate currents for the excessive APD lengthening and EAD promotion under other conditions, such as in type 3 long QT syndrome [13], [39]. The presence of late I_Na_, combined with window I_Ca,L_, helps the formation of the quasi-equilibrium states at the plateau voltage, which may be responsible for the underlying mechanism of late I_Na_ causing EADs. In addition, window I_Na_ alone may be strong enough to result in quasi-equilibrium states at lower voltages, especially when outward current is reduced.

To generate an ultralong APD, a slowly activating outward current is required to maintain the voltage at the plateau phase long enough but can eventually repolarize the cell. The delayed rectifier K current is the candidate current which has two components [24], the fast one, I_Kr_, and the slow one, I_Ks_. The slow activation time constant of I_Ks_ is longer than 2 s at −20 mV [25], about 4 times of the value in the LR1 model (shown in Fig. 6D). Reduction or elimination of I_Ks_ and I_Kr_ is known to be the causes of type 1 and type 2 long QT syndrome [3], respectively. Since the activation of I_Kr_ is fast, thus I_Ks_ is required for the ultralong APD to occur. In the absence of I_Ks_, blocking I_Kr_ or increasing inward currents may fail to generate ultralong APD but instead cause all-or-none repolarization failure. As shown in Figure 3, the 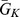 range exhibiting ultralong APD is much narrower for faster activation speed (compare Fig. 3A to Fig. 3D), and this range will be further narrowed if the activation speed is increased (γ<1).

A surprising observation in this study is that increasing inward currents (by either increasing the pedestal current or shifting the inactivation curve) can either lengthen or shorten APD (Figs. 4 and 5), depending on the control conditions. The cause is that increasing inward current increases the plateau voltage which in turn activates more I_Ks_ due to a faster activation speed and a larger steady-state open probability at a higher voltage (Fig. 6D) [25]. This same effect was also observed in a recent study by Sarkar and Sobie [35] that a greater I_Ca,L_ results in a smaller APD increase responding to I_Kr_ blockade. Based on the observation that increasing the conductance of inward currents can shorten APD, one would expect that decreasing the conductance of an outward current may not always increase APD, but can decrease APD under certain conditions. A well known example is that increasing the outward transient K current increases APD, and promotes EADs and APD alternans [40], [41], [42], [43].

In both experimental and simulation studies [44], [45], [46], [47], APD alternans is usually induced at fast heart rates. Here we show APD alternans can be induced in very slow heart rates. The mechanism of APD alternans is still related to steep APD restitution (see Fig. 7), but the ionic mechanisms are different. The APD alternans occurring in fast heart rates is mainly caused by I_Ca,L_ recovery from inactivation that results in steep APD restitution curves [45], [46]. Here the sensitive rate-dependence is caused by the window or pedestal I_Ca,L_ interacting with I_Ks_ recovery. This mechanism may be responsible for T-wave alternans observed clinically in long QT syndrome at normal or slow heart rates [22], [23].

Finally, the implications of our present study to the experimental observations shown in Figure 1 can be understood as follows. Based on the observations shown in Figure 3, the following possibilities can occur. The first one is that there is no or less window I_Ca,L_ in the epicardial and endocardial cells. After I_Kr_ is blocked, although APD is still lengthened, it cannot reach very long as in the M-cells. The second one is that the three types of cells have the same window I_Ca,L_. Since I_Ks_ is smaller in the M-cells [4], [5], after I_Kr_ is blocked, I_Ks_ is still large enough in the epicardial and endocardial cells to prevent the occurrence of long APD, but not for the M-cells. The lack of EADs in the M-cells, even though the APD is as long as 2 s, may be caused by the kinetics of I_Ca,L_, such as reduced slope of the steady-state inactivation curve (as the case shown in Fig. 7E).

### Limitations

A major limitation of the present study is the use of the LR1 model which lacks many of the details of the ionic currents and Ca cycling of a cardiac myocyte. For example, in real myocytes, LCC inactivation is regulated by both voltage and Ca [29]. Reducing or eliminating the Ca-dependent inactivation alters the steady-state inactivation curve of LCC and thus the window and pedestal I_Ca,L_, which then affects APD and EADs, such as the effects caused by CaM_1234_ [7], [8]. However, by altering the inactivation properties phenomenologically in the simplified model, we can still gain general mechanistic insights into why CaM_1234_ overexpression causes ultralong APD but not EADs. Another effect of Ca cycling is that spontaneous Ca release or Ca oscillations may promote EADs via Na-Ca exchange current [48], [49], which is a different mechanism of EADs and cannot be explained using the quasi-steady state analysis shown in Figure 6. In addition, the delayed rectifier K current has a fast component (I_Kr_) and a slow component (I_Ks_), while only a single one is present in the LR1 model, which may limit the physiological relevance of the predictions in this study. However, since the one in the LR1 model is equivalent to the slow component, one may consider this as the case in which I_Kr_ is blocked, such as in type 2 long QT syndrome. Nevertheless, the detailed physiological processes (or entities) that are responsible for these phenomena may be different under different conditions, but the underlying general dynamical mechanisms may be the same. The advantage of using the LR1 model is that we can alter the different properties of the ionic currents independently (as shown in Fig. 2) and thus study their effects on action potential properties, which may be difficult to realize in more detailed models. Moreover, the purpose of the present study is to investigate the general mechanisms and the necessary ionic determinants for the formation of ultralong APD and its sensitive slow rate-dependence and APD alternans at slow heart rates. The mechanisms and predictions obtained in this study need to be further validated in more detailed models and in experiments, and the effects on arrhythmogenesis need to be studied in tissue models.
